# The Origins of the Obesity Epidemic in the USA–Lessons for Today

**DOI:** 10.3390/nu14204253

**Published:** 2022-10-12

**Authors:** Norman J. Temple

**Affiliations:** Centre for Science, Athabasca University, Athabasca, AB T9S 3A3, Canada; normant@athabascau.ca

**Keywords:** food prices, obesity, sugar, sugar-sweetened beverages, fast foods

## Abstract

The obesity epidemic appeared in the USA in 1976–1980 and then spread across Westernized countries. This paper examines the most likely causes of the epidemic in the USA. An explanation must be consistent with the emergence of the epidemic in both genders and in all age groups and ethnicities at about the same time, and with a steady rise in the prevalence of obesity until at least 2016. The cause is closely related to changes in the American diet. There is little association with changes in the intake of fat and carbohydrate. This paper presents the opinion that the factor most closely linked to the epidemic is ultra-processed foods (UPFs) (i.e., foods with a high content of calories, salt, sugar, and fat but with very little whole foods). Of particular importance is sugar intake, especially sugar-sweetened beverages (SSBs). There is strong evidence that consumption of SSBs leads to higher energy intake and more weight gain. A similar pattern is also seen with other UPFs. Factors that probably contributed to the increased intake of UPFs include their relatively low price and the increased popularity of fast-food restaurants. Other related topics discussed include: (1) the possible importance of Farm Bills implemented by the US Department of Agriculture; (2) areas where further research is needed; (3) health hazards linked to UPFs; and (4) the need for public health measures to reduce intake of UPFs.

## 1. Introduction

Many thousands of research studies have been carried out on the causes of obesity and effective forms of treatment [1,2]. However, there is still a great deal to be learned. Starting around 1980, an obesity epidemic emerged that spread across the Western world. There has been much speculation as to the true cause of this epidemic but, as yet, no explanation has gained wide acceptance. This paper examines the evidence in an attempt to identify which factors are most likely to be responsible. The major focus is on the USA. The findings are potentially important as by identifying the major factors that triggered the obesity epidemic, this may suggest how obesity can be prevented.

## 2. Emergence of the Epidemic

The obesity epidemic emerged in Westernized countries during the 1980s [3,4]. Our best evidence is that this major event started a few years earlier in the USA, namely in 1976–1980 [5,6]. Obesity is defined as a BMI ≥ 30. There was only a small rise (approximately 0.5%) in the prevalence of obesity among American adults in the years 1971–1974 to 1976–1980, but this was followed by a rapid rise that continued for at least 20 years. The prevalence of obesity in American adults (age 20–74, both genders) rose from 15.0% in 1976–1980, to 23.3% in 1988–1994, and to 30.9% in 1999–2000. What is especially noteworthy is that this fast-rising prevalence among American adults was similar in all age groups, in both men and women, and in all major ethnic groups including Caucasian Americans, African Americans, and Mexican Americans [6]. This rise in the prevalence of obesity continued in both genders during the period 1999 to 2016 [7].

The data from the USA come from regular surveys that assess the health and nutritional status of adults and children. These surveys are known as the National Health and Nutrition Examination Survey (NHANES). They are carried out by the National Center for Health Statistics (NCHS), which is part of the Centers for Disease Control and Prevention (CDC).

## 3. Possible Causes of the Epidemic

Based on the information stated above, an explanation for the epidemic must be consistent with the following:

- The appearance of the epidemic in the USA in the years 1976–1980;
- Its appearance in both genders and in all age groups and ethnicities at about the same time;
- A steady increase in the prevalence of obesity that continued until at least 2016.

Where information was needed on particular topics, a literature search was carried out using the PubMed database. As this paper is an opinion piece rather than a review, I have been very selective regarding what information is included or excluded.

Several factors can be immediately eliminated as they do not act across the population in a mere five years or so. This includes genetics and epigenetics. The latter factors are certainly of much relevance in helping to explain why one person develops obesity but not another person who leads a similar lifestyle. However, the distribution of these factors is fairly stable across the population and for that reason cannot explain the sudden emergence of the obesity epidemic. Similarly, we can exclude maternal factors that act before birth or in early infancy. As with genetics these factors are certainly relevant in explaining person-to-person variation in the development of obesity. However, these factors cannot explain why the obesity epidemic appeared in different age groups at around the same time.

A reduction in physical activity seems to have played, at most, only a minor role [3]. Manual labor has steadily decreased in the USA over many decades. A significant decrease was seen during the 1960s and 1970s, but this shows only a weak association with the rapid increase in obesity that started in the late 1970s [3]. Moreover, the magnitude of the decrease in work-related physical activity is too small to have been an important factor in the increase in obesity. Another argument against a significant role for the decrease in work-related physical activity is that the rise in obesity was also seen among persons who were not of working age (i.e., those aged under 18 or over 65). Over the decades, the need for physical activity also steadily fell outside of work. In particular, the population had greater access to cars and public transport.

Finally, it is noteworthy that there is no evidence indicating a fall in leisure-time physical activity at around the time that the obesity epidemic started. Indeed, the late 1970s and 1980s was when exercise became more popular (e.g., jogging).

Obesity occurs when energy intake (food) is substantially higher than energy expenditure (in particular physical activity). The cause of the obesity epidemic must therefore be due to either an increase in energy intake from food or a decrease in physical activity (or a combination). As noted above it is unlikely that a decrease in physical activity played a significant role in the epidemic. From this, we can assume that the most likely explanation for the epidemic is changes in the American diet. A large body of evidence has linked the diet with body weight and risk of excess weight gain. Many changes have taken place in the American diet over the past several decades. We now explore how changes in the American diet may have led to the obesity epidemic.

### 3.1. Dietary Fat

A hypothesis that gained wide support during the 1970s was that the diets commonly eaten in the USA and across the Western world had an excessively high content of fat and that this played a major role in various chronic diseases of lifestyle. This hypothesis was extended to obesity. In 1977, the Senate Select Committee on Nutrition and Human Needs translated this hypothesis into actual policy with the publication of *Dietary Goals for the United States* [8,9]. One of the recommendations in this report was that Americans should reduce the fat content of their diet. This advice was repeated in 1980 with the publication of the first edition of *Dietary Guidelines for Americans* [10]. Recommendations for a reduced intake of fat steadily permeated to the general population [11]. The food industry responded by marketing such foods as lean meat and low-fat milk.

Despite these various actions it is unclear if there was an actual fall in fat intake after 1980. Findings from NHANES surveys (of 8600–10,000 persons) suggest that American adults reduced their fat intake as a proportion of energy during the years 1976–1980 to 1999–2000 (36.7% to 32.3% in men; 36.0% to 32.4% in women) [12]. However, there was a significant rise in energy intake at the same time. The net effect is that the *quantity* of fat in the diet appears to have risen slightly.

Another study used the findings from the Nationwide Food Consumption Survey (NFCS) of >10,000 American adults [13,14]. The data cover the period from 1977–1978 to 1987–1988. Fat intake as a proportion of energy fell from 41.0% to 36.6%. There was also a modest (approximately 4%) *fall* in energy intake. The net result is an approximate 14% fall in the *quantity* of fat in the diet. These findings must be viewed with some suspicion as they indicate that energy intake was falling while the prevalence of obesity was increasing.

The findings regarding changes in intake of fat and energy lack consistency between the above two studies. As the NFCS data may be unreliable, more reliance should be placed on the NHANES data. This suggests, therefore, that the fat intake of American adults was quite stable during the years 1976–1980 to 1999–2000. The findings from randomized controlled studies (RCTs) show that small changes in fat intake have little impact on body weight [15]. Taking these findings as a whole, we can conclude that there is, at most, only a very small relationship between changes in the fat content of the American diet and the appearance of the obesity epidemic.

As stated above the findings from NHANES indicate that there was a significant rise in energy intake at the same time as the fat intake of American adults was quite stable (1976–1980 to 1999–2000). This suggests that there was an increase in the *quantity* of carbohydrates in the diet. Many investigators have been quick to conclude that this increase in dietary carbohydrates was an important factor in the obesity epidemic [16]. However, that is a serious mistake as it ignores the major differences between different food sources of carbohydrates. When attention is turned away from the *quantity* of carbohydrate in the diet, and to the *nature of the foods* being eaten, the probable cause of the epidemic starts to emerge.

### 3.2. Sugar and Sugar-Sweetened Beverages

Sugar intake in the USA was fairly stable in the 1970s but then rose sharply after 1978. Per capita intake of total caloric sweeteners was 124.6 pounds in 1978, 132.7 pounds in 1988, and 154.1 pounds in 1997 [17]. This indicates an increase of 36.7 g/day (147 kcal/day) between 1978 and 1997. The increase in energy intake is sufficiently high to explain the increase in the average weight of the population [18]. One important factor that brought about the growth in sugar intake was a major drop in the price of sugar that occurred after 1980 [19].

The above data refers to total caloric sweeteners. However, there was a large shift from sucrose to high-fructose corn syrup, but there is no hard evidence that this is a significant factor in the obesity epidemic [20].

Surveys of the American diet document a large rise in the consumption of sugar-sweetened beverages (SSBs) during the time period 1977–1978 to 1994–1996 [21]. This accounts for much of the increase in sugar intake. During this time period the proportion of energy obtained from soft drinks increased from 4.1% to 7.0% at age 19–39 and from 1.9% to 4.0% at age 40–59.

RCTs and prospective cohort studies have generated strong evidence that consumption of SSBs leads to higher energy intake and more weight gain [22,23]. Findings from RCTs have reported that the addition of SSBs to the diet of adults leads to a 0.85 kg increase in body weight [22]. Findings from cohort studies indicate that an extra serving per day of SSB is associated with an increase in body weight in adults of around 0.12 to 0.22 kg [22]. This is probably also the case in the area of sugar intake in general; however, there are far fewer research studies [23]. This evidence strongly suggests that SSBs are a major factor responsible for the obesity epidemic.

### 3.3. Ultra-Processed Foods

SSBs are a type of food commonly referred to as ultra-processed foods (UPFs) [24]. UPFs are typically prepared from mostly cheap sources of dietary energy and nutrients plus additives. They are mostly high in calories, salt, sugar, and fat but contain minimal amounts of whole foods. As a result, they have a low content of dietary fiber, phytochemicals (for example, lutein, lycopene, and anthocyanins), and of various micronutrients, such as potassium, magnesium, and vitamin C. Examples of UPFs include white bread, sweetened breakfast cereals, cookies, savory snacks, cakes, candy, ice cream, margarine, sausages, and pizza.

There is impressive evidence that UPFs play a major role in the obesity epidemic [25]. The above-mentioned surveys of the American diet that documented a large rise in the consumption of SSBs during the time period 1977–1978 to 1994–1996 also revealed a similar increase in the intake of other UPFs [21]. The largest increases were seen for salty snacks and pizza. During this time period, the proportion of energy obtained from various types of UPFs in the age group 40–59 increased from 1.4% to 3.8% for salty snacks, from 0.5% to 1.7% for pizza, from 0.5% to 1.4% for candy, and from 1.2% to 1.6% for French fries.

The following RCT demonstrates the potential of UPFs to cause one to be overweight and obese [26]. Subjects were fed two nutritionally similar diets for 2 weeks. The diets differed only in their degree of processing: one comprised UPFs while the other was based on minimally processed foods. Subjects were instructed to consume as much or as little as desired. The UPF diet resulted in, on average, a 508 kcal/day higher energy intake than the other diet. It also resulted in a commensurate amount of weight gain (an average of 0.9 kg).

These findings are consistent with those from prospective cohort studies. The combined results of three cohort studies conducted on 121,000 men and women in the USA reported a positive association of weight change with intake of potato chips (a UPF) but a negative association with intake of several minimally processed foods, namely fruits, vegetables, whole grains, and nuts [27]. Findings from cross-sectional studies (analyzed as part of a systematic review and meta-analysis) indicate that that a relatively high consumption of UPFs is associated with a 39% higher risk of overweight/obesity and of high waist circumference [28]. Another systematic review and meta-analysis of observational studies also concluded that there is a strong association between the intake of UPFs and risk of overweight and obesity [29].

One factor that may have been driving the upward consumption of UPFs is the strong trend towards eating at fast-food restaurants. The surveys of the American diet that were discussed earlier reported that the proportion of energy in the American diet that was obtained from eating out, including at fast-food restaurants, went from 9.4% to 21.3% during the years 1977–1978 to 1994–1996 [19]. Fast-food restaurants may be of particular importance as most of the food sold there is UPF (e.g., burger, fries, and a cola drink at McDonald’s). Evidence from prospective cohort studies have reported that more frequent eating at these restaurants is associated with a greater increase in body weight and of waist circumference [30].

### 3.4. The Role of Farm Bills and Food Prices

Analyses of food prices has clearly shown that healthier diets cost significantly more than less healthy diets. For example, an international study compared the cost of healthy vs. less healthy food-based diet patterns. The researchers concluded that the top (most healthy) quantile cost $1.54 more per 2000 kcal (based on 2011 prices) [31]. It is well established that shoppers tend to buy less expensive foods. As UPFs are a feature of less healthy diets, shoppers will therefore frequently select these foods. These findings strongly indicate, therefore, that relatively low food prices encourage the general population to eat UPFs which, in turn, play a major role in obesity.

It has been suggested many times that a key event leading to the supply of large quantities of UPFs at relatively low prices was a major policy change by the Nixon administration in the early 1970s. Farm Bills implemented by the US Department of Agriculture (USDA) gave increased subsidies to farmers. This may have directly led to a large increase in the supply of agricultural products that food manufacturers then turned into UPFs which were sold at a relatively low price. This triggered and has sustained the obesity epidemic. This hypothesis has been the subject of much investigation, but no firm conclusions have been reached [31,32].

## 4. Research Challenges

The most plausible explanation for the obesity epidemic in the USA is that around 1978–1980 there was a major increase in the consumption of UPFs. However, this hypothesis is still far from proven. There is a need for further research in the following key areas:

1. The change in intake of different types of food in the American diet, especially after 1978. In particular, information is needed on the changing intake of different types of UPFs in different sections of the US population from the 1970s to the present;
2. The factors that explain the increase in intake of UPFs (e.g., subsidies given to farmers by the USDA, changing food prices, and trends in the number of meals purchased at fast-food restaurants);
3. Changes in energy intake across the population and whether this is consistent with increases in the prevalence of obesity;
4. Whether findings from the US population also explain the obesity epidemic that occurred in other Westernized countries;
5. How different types of UPFs affect energy intake and lead to weight gain. A RCT that investigated this was described earlier [26]. However, while highly informative, its duration was only 2 weeks;
6. The most effective means to reduce the prevalence of obesity by reducing intake of UPFs across the population;
7. To determine whether a diet with a low content of UPFs is effective in aiding weight loss.

## 5. A Brief Overview of Ultra-Processed Foods and Health

The evidence presented in this paper strongly suggests that UPFs are a major factor responsible for the obesity epidemic. However, the negative impact of UPFs on health extends well beyond obesity. Observational evidence has revealed a clear association between the intake of UPFs and several disorders including cancer [33], type 2 diabetes [34], cardiovascular disease [28], and depression [28]. A recent systematic review and meta-analysis of 40 prospective cohort studies concluded that high consumption of UPFs (comparing highest vs. lowest intake groups) is associated with a 29% higher all-cause mortality [35].

UPFs are certain to have a major negative impact on the nutritional status of American adults as they comprise an estimated 57.0% of energy intake whereas minimally processed foods represent only 27.4% [36]. These data are from the NHANES survey in 2017–2018. As mentioned earlier, this will inevitably result in the diet having a low content of dietary fiber, phytochemicals, and of various micronutrients.

## 6. Ultra-Processed Foods and Public Health: The Need for Serious Action

These findings compel the conclusion that priority must be given to efforts that will reduce the consumption of UPFs, both in the USA and internationally. One strategy is a health education approach. Nutrition advice is delivered to the general public in diverse ways. Of particular importance governments deliver nutrition advice by way of food guides. These need to explicitly emphasize the importance of reducing the intake of UPFs. Similarly, the addition of labels to the front of food packages may help shoppers make healthier food choices [37]. Warning labels is one design that shows much promise (e.g., warning shoppers that a food has a high content of sugar).

Unfortunately, much evidence clearly demonstrates that delivering nutrition advice to the population achieves only modest success. A much more effective strategy is one based on policies implemented by governments that impose regulations on various organizations including food companies [38]. For example, schools can be ordered to cease selling UPFs in vending machines. Similarly, the amount of UPFs in school meals should be minimized.

Manipulation of food price is another policy approach that has much potential. It is well established that an increase in price leads to a decrease in sales (and vice versa). This is known as price elasticity. Accordingly, taxes and subsidies can be used in order to achieve a desired effect on food consumption [39,40,41]. More specifically, the price of UPFs, especially sugar-rich foods, can be increased while the price of healthy foods, such as fruit, vegetables, and whole grains, can be reduced.

It is very probable that policies implemented by governments are not only more effective than those based on nutrition education, but they are also much more cost-effective. This strategy has a long history and has achieved great success. Prominent examples include providing the population with safe drinking water, the addition of various micronutrients to particular foods, and the removal of *trans* fatty acids from foods [39].

## 7. Conclusions

The evidence presented here supports the case that the large quantities of UPFs in the diet have played a dominant role in the obesity epidemic. While the main focus here has been on the situation in the USA, it seems highly likely that UPFs are also heavily involved in the epidemic of obesity that has spread across the Western world, starting around 1980. However, there are still many gaps in the evidence and much more research is therefore needed.

A high dietary intake of UPFs is closely associated with a range of damaging effects on health in addition to obesity. For that reason, dietary advice should emphasize a reduction in the intake of UPFs across the population. While health education, such as food guides and food labels, is a useful way to work towards that goal, much evidence indicates this strategy achieves only limited success. A more effective strategy is one based on policies and regulations implemented by governments. Examples of such policies include removing UPFs from school meals, increasing the price of SSBs by adding a tax, and using subsidies to reduce the price of fruit and vegetables.

## Data Availability

Not applicable.
